# Mathematical Model and Calibration Experiment of a Large Measurement Range Flexible Joints 6-UPUR Six-Axis Force Sensor

**DOI:** 10.3390/s16081271

**Published:** 2016-08-11

**Authors:** Yanzhi Zhao, Caifeng Zhang, Dan Zhang, Zhongpan Shi, Tieshi Zhao

**Affiliations:** 1 Key Laboratory of Parallel Robot and Mechatronic System of Hebei Province, Yanshan University, Qinhuangdao 066004, China; cfzhang@stumail.ysu.edu.cn (C.Z.); liyang@stumail.ysu.edu.cn (Z.S.); tszhao@ysu.edu.cn (T.Z.); 2 Key Laboratory of Advanced Forging & Stamping Technology and Science of Ministry of Education of China, Yanshan University, Qinhuangdao 066004, China; 3 Department of Mechanical Engineering, Lassonde School of Engineering, York University, 4700 Keele Street, Toronto, ON M3J1P3, Canada; dan.zhang@lassonde.yorku.ca

**Keywords:** six-axis force sensor, parallel mechanism, flexible joints, calibration experiment

## Abstract

Nowadays improving the accuracy and enlarging the measuring range of six-axis force sensors for wider applications in aircraft landing, rocket thrust, and spacecraft docking testing experiments has become an urgent objective. However, it is still difficult to achieve high accuracy and large measuring range with traditional parallel six-axis force sensors due to the influence of the gap and friction of the joints. Therefore, to overcome the mentioned limitations, this paper proposed a 6-Universal-Prismatic-Universal-Revolute (UPUR) joints parallel mechanism with flexible joints to develop a large measurement range six-axis force sensor. The structural characteristics of the sensor are analyzed in comparison with traditional parallel sensor based on the Stewart platform. The force transfer relation of the sensor is deduced, and the force Jacobian matrix is obtained using screw theory in two cases of the ideal state and the state of flexibility of each flexible joint is considered. The prototype and loading calibration system are designed and developed. The K value method and least squares method are used to process experimental data, and in errors of kind Ι and kind II linearity are obtained. The experimental results show that the calibration error of the K value method is more than 13.4%, and the calibration error of the least squares method is 2.67%. The experimental results prove the feasibility of the sensor and the correctness of the theoretical analysis which are expected to be adopted in practical applications.

## 1. Introduction

With the ability of measuring three force components and three torque components, the six-axis force sensor is one kind of the most important and challenging sensors, used widely in many research areas such as wind tunnel balances, thrust stand testing of rocket engines, and in robotics, the automobile industry, aeronautics, etc. [1]. Compared with single-axis sensors, not only the volumes and prices of multi-axis force sensors are considered, but also their structures, in order to achieve a balance between the isotropy of force/torque and that of sensitivity [2]. Recently, researchers all over the world have done a lot of work on six-axis force sensors. 

Since the Stewart platform was applied to the measurement of space six-axis forces by measuring the forces in the six legs with convection elements in 1983 [3], parallel structure have been widely used in six-axis force sensors [4], stimulated by the advantages of good stiffness, symmetric and compact structure, and straightforward mapping expression between the wrenches applied on the platform and the measured leg forces. Nguyen et al. [5] developed a Stewart platform-based sensor with LVDT’s mounted along the legs for force/torque measurement in the presence of passive compliance. Durand changed the traditional Stewart structure and put piezoelectric quartz inside the Stewart structure and pre-tightened it [6], therefore, the sensor was more compact and could be used to measure tensions. Dwarakanath et al. [7,8] introduced the usage of ring-shaped sensing element in the Stewart platform sensor, and presented a simply supported, ‘joint less’ six-axis parallel force sensor. Kim et al. [9] put forward a six-axis wrist force sensor using FEM for an intelligent robot. 

Although research on the traditional Stewart platform-based six-axis force sensor is quite mature, the sensor with common joints has a lower precision, and the performance of each direction is different. In comparison, the sensor with flexible joints has features of compact structure, fast response, small accumulated error, no mechanical friction, and high measurement accuracy because of the use of integrated design, so it has broad application prospects [10,11,12,13,14]. Kerr [15] suggested that the Stewart platform with instrumented elastic legs can be used as a six-axis force sensor. Jin et al. [16] proposed a novel isotropic six-axis force sensor based on a variation of Stewart platform, whose three pairs of elastic legs are perpendicular to the three orthogonal surfaces of the basic cube. Gao et al. [17] developed a six-axis controller based on Stewart platform-based force sensor, and introduced the elastic joints to replace the real spherical joints which made the miniaturization possible. Liang et al. [18] designed and developed a new six-axis sensor system with a compact monolithic elastic element (EE), which detected the tangential cutting forces **F_x_**, **F_y_** and **F_z_** (i.e., forces along x-, y-, and z-axis) as well as the cutting moments **M_x_**, **M_y_** and **M_z_** (i.e., moments about x-, y-, and z-axis) simultaneously. Unfortunately, most of the sensors mentioned above are used in a small range of applications. Nowadays, the large measurement range six-axis force sensor is more and more widely needed in applications such as aircraft landing gear momentum tests, rocket thrust tests, spacecraft docking and wind tunnel tests, whose precision requirements are also getting increasingly higher. However, when the parallel six-axis force sensor is used in large measurement range occasions, the traditional joints are difficult to be processed into flexible joints, and the improvement of measurement accuracy will be affected, so parallel six-axis force sensors with large measurement range and high accuracy are urgently required.

When the force transmission relation of the sensor is established, the stiffness of each flexible joint and the whole stiffness which has a direct impact on the measuring accuracy, bearing capacity and dynamic performance, and is one of the important indexes to measure the performance of sensors must be considered. Research on stiffness of the sensors based on parallel mechanism began in the 1990s [19], which gave Gosselin’s minimalist stiffness mapping model of parallel mechanism under no loading. Then, Griffis and Duffy [20] proposed a kind of parallel mechanism with branches which are assumed to be wire springs. Under the premise of joints are equivalent, they considered the effect of differential motion, and established the complete stiffness model by the analytical method. Chakarov [21] analyzed the effect of external load on the overall stiffness matrix in force redundant parallel mechanism. Pashkevich et al. [22] proposed a kind stiffness modeling method of the over-constrained parallel mechanism with flexible branches and flexible drive joints. Zhang and Wei [23] derived the stiffness model of the mechanism and evaluated the global stiffness using the sum of the diagonal elements of the stiffness matrix. However, existing institutions to complete stiffness modeling methods are very complex, and there is little research combining together stiffness and force Jacobian matrix.

In this paper, a kind of six-axis force sensor based on 6-UPUR parallel mechanism with flexible joints, which has large measurement range and high accuracy is proposed. Meanwhile, the complete mathematical model considering the flexibility of the joints is established, and the calibration experiment is completed. 

The structure of this paper is as follows: after the introduction, Section 2 concerns the structural analysis of the sensors, including the intrinsic disadvantages of the traditional parallel sensors based on the Stewart platform and the structure features of the large measurement range six-axis force sensor of 6-UPUR parallel mechanism with flexible joints. The measuring principle, mathematical model of the structure which included the ideal state and the state of flexibility of each flexible joint is considered in Section 3. Section 4 introduces the experimental research on static calibration of the sensor prototype. Section 5 presents the results of the experiment. The paper is concluded in Section 6, summarizing the work that has been done.

## 2. Sensor Structure

**Figure 1:**
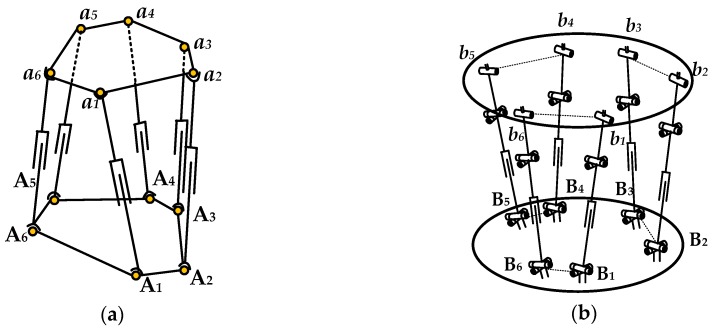
(**a**) The force sensor structure based on the traditional Stewart platform; (**b**) the force sensor structure based on 6-UPUR parallel mechanism.

Figure 1a illustrates the traditional parallel sensor diagram, which is based on the Stewart platform and composed of a measuring platform, a lower fixed platform and six elastic legs connecting the two platforms with traditional spherical joints. 

Considering the traditional spherical joints are difficult to processed into flexible joints in a large measurement range situation, and the measurement accuracy will be affected, which restricts its application in six-axis force sensors based on flexible parallel mechanism, this paper proposes a kind of structure model of six-axis force sensor based on a parallel 6-UPUR mechanism. As shown in Figure 1b, it consists of a measuring platform, a fixed platform and six measuring legs divided into three groups of legs and two legs in each group are located in a vertical plane. Each measuring leg contains a single-axis force sensor and connects the fixed platform with flexible universal joint, and connects the measuring platform with combined spherical joint. Through the improvement of the traditional Stewart platform mechanism, and the introduction of flexible joints which have the peculiarities of non-clearance, friction-less and high sensitivity to replace the traditional spherical joints, it is possible to develop a sensor with a large measurement range.

**Figure 2:**
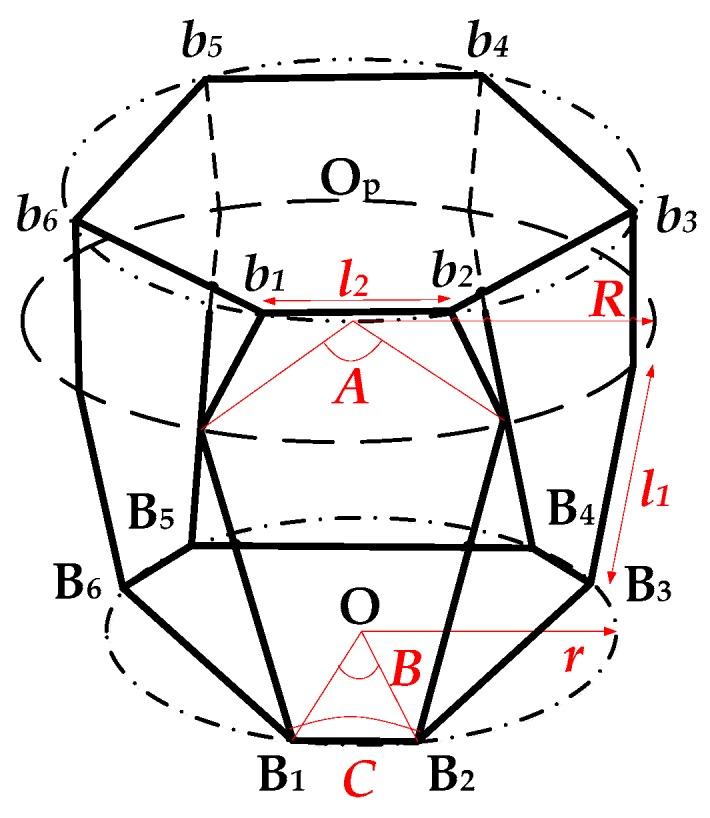
Schematic diagram of characteristic parameters.

The characteristic parameters of the parallel 6-UPUR six-axis force sensor include radius *R* of the measuring platform, radius *r* of the lower platform, the positioning angle *A*, *B*, *C*, the center distance *l_1_* of the U joint and location center distance *l_2_* of the R joint, as shown in Figure 2.

## 3. Mathematical Model

### 3.1. Ideal Mathematical Model

#### 3.1.1. Force Analysis

There are reacting forces on measuring legs when an external force loaded on the measuring platform of the parallel sensor whose surface is assumed to be friction-less and continuous, and the reacting force is considered along the axis of each measuring leg. According to the space static balance conditions of the measuring platform, the following equation can be obtained on screw theory [24]:

(1) $$\sum_{i=1}^{6}f_{i}S_{i}=F+\in M$$

where *f_i_* represents the reacting force on the measuring legs; *S_i_* represents the unit line vector along the ith measuring leg; ***F*** and ***M***, respectively, represent the ith loaded force vector and toque vector on the center of the measuring platform; ∈ is the dual sign.

Equation (1) can be rewritten in the form of matrix expression as:

(2) $$F_{\omega }=G_{f}^{F}f$$

where $F_{\omega }={\left[F_{x}\;F_{y}\;F_{z}\;M_{x}\;M_{y}\;M_{z}\right]}^{T}$ is the vector of six-axis external force applied on the measuring platform; $f={\left[f_{1}\;f_{2}\;f_{3}\;f_{4}\;f_{5}\;f_{6}\right]}^{T}$ is the vector composed of the reacting forces of the six measuring legs; $G_{f}^{F}$ is the first-order static influence coefficient matrix. If $G_{f}^{F}$ is non-singular matrix, the inverse mapping between ***F_ω_*** and ***f*** is:

(3) $$f=G_{F}^{f}F_{w}$$

where, $G_{F}^{f}={\left(G_{f}^{F}\right)}^{-1}$ is the force Jacobi matrix, and it is the mapping matrix from the external force loaded on the measuring platform of the sensor to the force produced on the six measuring leg. $G_{f}^{F}$ is closely related with the geometric structure of the sensor, and the characteristics of $G_{f}^{F}$ determine stiffness, isotropy, sensitivity and many other features of the sensor. 

#### 3.1.2. Ideal Mathematical Model

Each leg of the parallel 6-UPUR mechanism can be seen as a series open-chain mechanism which is composed of six links and six joints, and the U joint is equivalent to two joints whose axes are vertical and intersect. The base is called link 0, which is not included in the six links. The first link is connected to the base by the first rotational joint; the second link is connected to the first link by the second rotational joint, and so on. 

**Figure 3:**
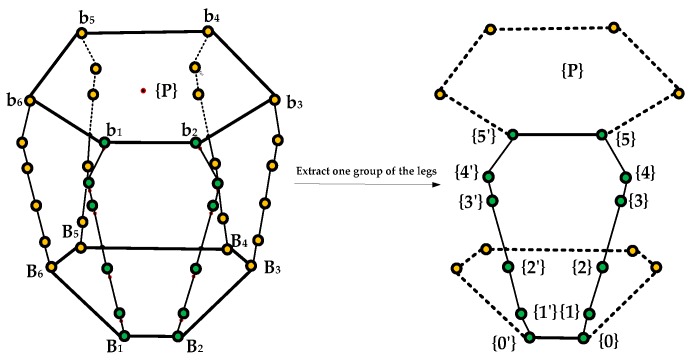
Schematic diagram of the links and joints.

The coordinate system attached to the base fixedly is referred to as {0}; the coordinate system on the reference point of the end of the leg is referred to as {P}; coordinate system attached to the ith link fixedly is referred to as{i}, as shown in Figure 3.

The differential motion vector of central reference point *P* which is on the measuring platform is:

(4) $$D_{P}={\left(\begin{array}{cc} \delta_{h} & d_{P} \end{array}\right)}^{T}=\left[G_{\phi }^{P}\right]\begin{array}{c} \dot{\phi } \end{array}$$

where: $\delta_{h}=\left[\delta_{hx}\;\delta_{hy}\;\delta_{hz}\right]$ is the angle variable, $d_{P}=\left(d_{P_{x}}\;d_{P_{y}}\;d_{P_{z}}\right)$ is the displacement variable, $G_{\phi }^{P}$ is the first-order static influence coefficient matrix, $\dot{\phi }=\;{\left[{\dot{\phi }}_{1}\ldots {\dot{\phi }}_{6}\right]}^{T}$ is the differential motion of the six legs:

(5) $$\left[G_{\phi }^{P}\right]=\left[\begin{array}{cccccc} S_{1} & S_{2} & 0 & S_{4} & S_{5} & S_{6}\\ S_{1}\times \left(P-R_{1}\right) & S_{2}\times \left(P-R_{2}\right) & S_{3} & S_{4}\times \left(P-R_{4}\right) & S_{5}\times \left(P-R_{5}\right) & S_{6}\times \left(P-R_{6}\right) \end{array}\right]$$

$S_{1}\ldots S_{6}$ are the directions of joints’ axes of each leg, and they are:

(6) $$S_{1}={\left\{\begin{array}{ccc} 0 & 0 & 1 \end{array}\right\}}^{T}\;S_{j}=T_{j-1}{\left[\begin{array}{ccc} 0 & -\sin\alpha_{\left(j-1\right)j} & \cos\alpha_{\left(j-1\right)j} \end{array}\right]}^{T}$$

*T_j_* is rotational transformation matrix:

(7) $$T_{j}=\left[\begin{array}{ccc} a_{jk} & S_{j}\times a_{jk} & S_{j} \end{array}\right]\left(j=1,2,\cdots ,6\right)$$

$a_{j\left(j+1\right)}$ is the direction of the common normal line between adjacent axes:

(8) $$a_{12}={\left[\begin{array}{ccc} \cos\theta_{1} & \sin\theta_{1} & 0 \end{array}\right]}^{T}\;a_{j\left(j+1\right)}=T_{j-1}{\left[\begin{array}{ccc} \cos\theta_{j} & \cos\alpha_{\left(j-1\right)j}\sin\theta_{j} & \sin\alpha_{\left(j-1\right)j}\sin\theta_{j} \end{array}\right]}^{T}\left(j=2,3,\cdots ,6\right)$$

(9) $$P-R_{n}=\sum_{r=n+1}^{6}\left(a_{\left(r-1\right)r}a_{\left(r-1\right)r}+d_{r}S_{r}\right)+T_{j}P^{\left(6\right)}$$

where $a_{\left(r-1\right)r}$ is length of the common normal line between adjacent axes, $d_{r}$ is offset along the axis of rotation. $P^{\left(6\right)}$ is the representation of the point *P* in coordinate at the end of the leg, and the left leg and the right leg are separately expressed as:

(10) $$P_{l}^{\left(6\right)}=\left[\begin{array}{ccc} -\sqrt{R^{2}-\frac{l_{2}^{2}}{4}} & \frac{l_{2}}{2}\cos\frac{C}{2} & -l_{2}\sin\frac{C}{2} \end{array}\right]\;P_{r}^{\left(6\right)}=\left[\begin{array}{ccc} -\sqrt{R^{2}-\frac{l_{2}^{2}}{4}} & -\frac{l_{2}}{2}\cos\frac{C}{2} & -l_{2}\sin\frac{C}{2} \end{array}\right]$$

where:

(11) $$C=\arccos\left[1-\frac{{\left(R\sqrt{1-\cos A}-r\sqrt{1-\cos B}\right)}^{2}}{l_{2}^{2}}\right]$$

According to the above equations, ${\left[G_{\phi }^{P}\right]}_{l}$ and ${\left[G_{\phi }^{P}\right]}_{r}$ can be obtained, separately:

(12) [GϕP]l=[001a5+ypa1+a4+d3+d6+zp0010xp0−(a4+d3+d6+zp)000100010xp0−(a4+d6+zp)001a5+ypd6+zp01000−xp−yp][GϕP]r=[001yp'−a5a1+a4+d3+d6+zp'00−10−xp'0a4+d3+d6+zp'0001000−10−xp'0a4+d6+zp'001yp'−a5d6+zp'01000−xp'−yp']

Because the *z* axis of the fixed coordinate system *O-xyz* which is on the center of the lower platform is the direction of *S*_1_, and selection of coordinate system has a direct impact on the first-order static influence coefficient matrix, so it is necessary to take the symmetry of the load platform structure into account, thus:

(13) $${}^{O}\left[G_{\phi }^{P}\right]{}_{i}=\left[\begin{array}{cc} {}_{O_{i}}^{O}R & 0\\ S{\left({}^{O}P{}_{O_{i}O}\right)}_{O_{i}}^{O}R & {}_{O_{i}}^{O}R \end{array}\right]{\left[G_{\phi }^{P}\right]}_{r}\left(i=1,3,5\right)\;{}^{O}\left[G_{\phi }^{P}\right]{}_{j}=\left[\begin{array}{cc} {}_{O_{i}}^{O}R & 0\\ S{\left({}^{O}P{}_{O_{i}O}\right)}_{O_{i}}^{O}R & {}_{O_{i}}^{O}R \end{array}\right]{\left[G_{\phi }^{P}\right]}_{l}\left(j=2,4,6\right)$$

When movement of the upper platform is known, movement of the joints in each leg can be written:

(14) $${\overset{•}{\phi }}^{\left(r\right)}={\left[G_{\phi }^{P}\right]}^{-1\left(r\right)}D_{P}\left(r=1,2,\cdots ,6\right)$$

When six active members of the mechanism are determined, equations of the six active movements from the above equations are taken out and expressed as:

(15) $$\begin{array}{l} {\overset{•}{\phi }}_{\alpha }^{\left(a\right)}={\left[G_{\phi }^{P}\right]}_{\alpha :}^{-1\left(a\right)}D_{P}\\ {\overset{•}{\phi }}_{\beta }^{\left(b\right)}={\left[G_{\phi }^{P}\right]}_{\beta :}^{-1\left(b\right)}D_{P}\\ \begin{array}{c} \vdots \end{array}\\ {\overset{•}{\phi }}_{\zeta }^{\left(f\right)}={\left[G_{\phi }^{P}\right]}_{\zeta :}^{-1\left(f\right)}D_{P} \end{array}$$

where ${\dot{\phi }}_{\alpha }^{\left(a\right)}$ is the differential motion of the six legs, its subscripts are leg’s number and joint’s number respectively, ${\left[G_{\phi }^{P}\right]}_{\alpha :}^{-1\left(a\right)}$ represents the *α-*th line of the inverse matrix ${\left[G_{\phi }^{P}\right]}^{-1\left(a\right)}$.

Combining the above six equations to constitute a matrix expression, like this: $\dot{q}=\left[G_{P}^{q}\right]D_{P}$. The inverse solution is $D_{P}=\left[G_{q}^{P}\right]\dot{q},\left[G_{q}^{P}\right]={\left[G_{P}^{q}\right]}^{-1}$.

Furthermore, the first-order static influence coefficient matrix can be obtained:

(16) $$G_{f}^{F}={\left({\left[\begin{array}{cccccc} {}^{O}\left[G_{\phi }^{P}\right]{}_{1}^{-1}\left(3,:\right) & {}^{O}\left[G_{\phi }^{P}\right]{}_{2}^{-1}\left(3,:\right) & {}^{O}\left[G_{\phi }^{P}\right]{}_{3}^{-1}\left(3,:\right) & {}^{O}\left[G_{\phi }^{P}\right]{}_{4}^{-1}\left(3,:\right) & {}^{O}\left[G_{\phi }^{P}\right]{}_{5}^{-1}\left(3,:\right) & {}^{O}\left[G_{\phi }^{P}\right]{}_{6}^{-1}\left(3,:\right) \end{array}\right]}^{T}\right)}^{-1}$$

where *^O^*${\left[G_{\phi }^{P}\right]}_{i}^{-1}\left(3,:\right)\left(i=1,\ldots ,6\right)$ represents the third row vectors of the matrix *^O^*${\left[G_{\phi }^{P}\right]}_{i}^{-1}\left(i=1,\ldots ,6\right)$.

Thus, the force Jacobi matrix is $G_{F}^{f}={\left[G_{f}^{F}\right]}^{-1}$, and that is the mapping matrix from the external force applied on the measuring platform of the sensor to the force produced on the six elastic legs in the ideal condition.

### 3.2. Mathematical Model of the Sensor with Flexible Joints 

**Figure 4:**
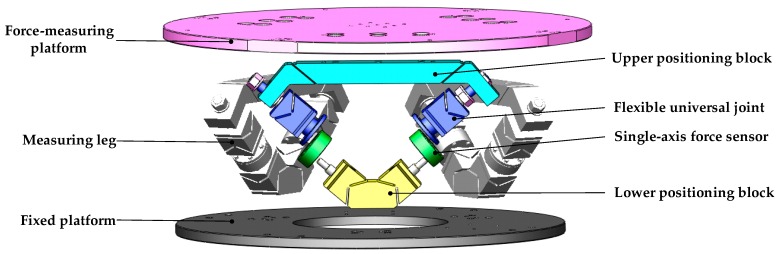
Diagram of the large measurement range force sensor of 6-UPUR parallel mechanism with flexible joints.

The six-axis force sensor adopts the structure that all joints are flexible joints with single degree of freedom, and its 3D model is shown in Figure 4. Each leg is a split structure. The lower part of the leg is composed of a flexible universal joint with an integral structure and a lower positioning block. Each elastic leg is connected to the measuring and fixed platforms through the upper and lower positioning blocks by bolts. Thus, it’s realized that the decomposition of the six-axis external force to the six legs. 

**Figure 5:**
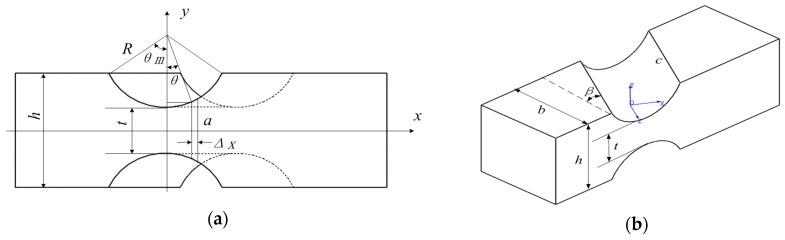
(**a**) The front view of flexible rotation joint; (**b**) the graphic model of flexible rotation joint.

The upper positioning block is composed of two flexible rotation joint, which form a flexible spherical joint with composite flexible universal joint by assembling relationship. Its front view and graphic model are shown in Figure 5. By using the knowledge of material mechanics, the rotational stiffness of the flexible rotary joint is obtained: *k*_1_ = 8.592 × 10^4^ (N·m/rad).

**Figure 6:**
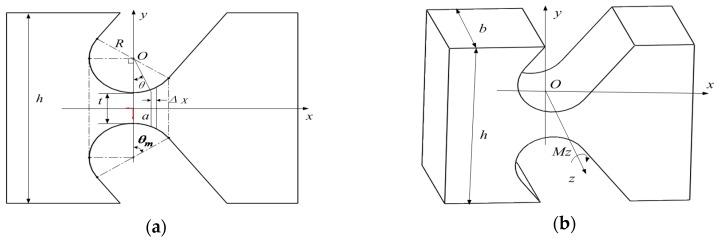
(**a**) The front view of a single joint; (**b**) the graphic model of a single joint.

The lower positioning block is an integral structure, which is composed of two symmetrical flexible universal joints, with the same structure as the flexible universal joint in the upper part of the leg. Its front view and graphic model of a single joint are shown in Figure 6. By using the knowledge of material mechanics, the rotational stiffness of a single joint is obtained: *k*_2_ = 4.0852 × 10^4^ (N·m/rad).

Considering the effect of elastic deformation of joints on $G_{F}^{f}$, so the following will re-establish the complete force Jacobian matrix. 

**Figure 7:**
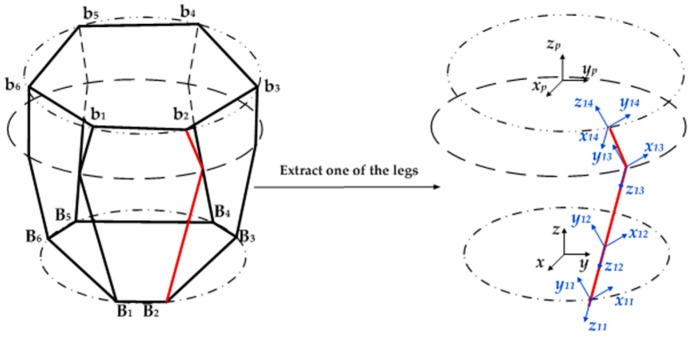
Schematic diagram of the coordinate systems for the flexible joints.

Figure 7 is the schematic diagram of flexible element coordinate systems’ establishment on a leg. where, *O-xyz* is the fixed coordinate system, *O_i1_-x_i1_y_i1_z_i1_, i =* 1,2,3,…,*6* is the local coordinate system which is established on the reference point of the lower positioning block, *O_i2_-x_i2_y_i2_z_i2_* is the local coordinate system which is established on the reference point of the standard one-axis force sensor, *O_i3_-x_i3_y_i3_z_i3_* is the local coordinate system which is established on the reference point of the flexible U joint, *O_i4_-x_i4_y_i4_z_i4_* is the local coordinate system which is established on the reference point of the integrated positioning block, *O_ip_-x_ip_y_ip_z_ip_* is the reference coordinate system which is established on the reference point of the end of a flexible series leg.

When there is the six-axis external force loaded on the end of the flexible series leg, by the principle of virtual work, the following equation can be obtained:

(17) $$\{\begin{array}{c} \Delta X_{j}=J_{j}X_{j}=\left[\Delta x_{j},\Delta y_{j},\Delta z_{j},\Delta \alpha_{x_{j}},\Delta \alpha_{y_{j}},\Delta \alpha_{z_{j}}\right]\\ F_{j}=J_{Fj}F \end{array}\left(j=1,2,3,4\right)$$

where Δ***X****j* is deformation vector of the end of leg’s reference point, ***X****j* is the elastic deformation vector of the end of the *j-*th basic flexible unit, ***J****_j_* is the pose transformation matrix, ***F****_j_* = [*f_x_*, *f_y_*, *f_z_*, *m_x_*, *m_y_*, *m_z_*]^T^ is the six-axis non-coplanar force, $F_{j}={\left[f_{x_{j}},f_{y_{j}},f_{z_{j}},m_{x_{j}},m_{y_{j}},m_{z_{j}}\right]}^{T}$ is the reaction force on the end of the *j-*th basic flexible unit, ***J****_Fj_* is the force transformation matrix.

According to the deformation superposition principle of series leg, the total deformation vector of the end of flexible series leg’s reference point caused by movement and rotation deformation vector of every basic flexible unit can be obtained:

(18) $$X=\sum_{j=1}^{4}\Delta X_{j}=\sum_{j=1}^{4}J_{j}X_{j}=J_{1}X_{1}+J_{2}X_{2}+J_{3}X_{3}+J_{4}X_{4}$$

It can be known by the definition of the stiffness matrix, the relationship between the six-axis external force at the end of the leg reference point and the end deformation of the leg is:

(19) F=KX

where ***K*** is the stiffness matrix of the end of flexible series leg.

**Figure 8:**
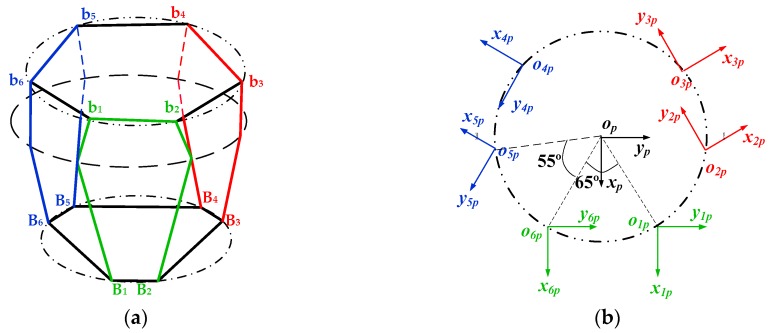
Schematic diagram of flexible element coordinate systems’ establishment: (**a**) The structure diagram; (**b**) Top view.

Substituting Equations (17) and (20) into Equation (21), the following equation can be obtained:

(20) $$X=K^{-1}F=\sum_{j=1}^{4}J_{j}X_{j}=\sum_{j=1}^{4}J_{j}K_{j}^{-1}F_{j}=\sum_{j=1}^{4}J_{j}K_{j}^{-1}J_{Fj}F$$

where ***K****_j_* (*j* = 1,2,…,4) is the stiffness matrix of the *j*th basic flexible element. The end reference point and the coordinate system of each flexible series leg are established as shown in Figure 8. *O_p_-x_p_y_p_z_p_* is the reference coordinate system established on the center point of the upper platform. *O_ip_-x_ip_y_ip_z_ip_*, *i* = 1,2,…, 6 is the reference coordinate system which is established on the reference point of the end of the flexible series leg.

On the assumption that the stiffness of the upper platform and all of branches is infinite, neglecting the minor deformation caused by force and the thickness of upper platform, when there is a six-axis external force, the geometric compatibility conditions between the end reference point and the reference point of the upper platform of the *i*th flexible series leg:

(21) $$\Delta X=\left[\begin{array}{c} \begin{array}{c} \begin{array}{c} \begin{array}{c} \Delta x\\ \Delta y \end{array}\\ \Delta z\\ \Delta \alpha_{x} \end{array}\\ \Delta \alpha_{y} \end{array}\\ \Delta \alpha_{z} \end{array}\right]=\left[\begin{array}{cc} {}_{O_{ip}}^{O_{p}}R^{T} & -{}_{O_{ip}}^{O_{p}}R^{T}S\left(r_{i}\right)\\ 0_{3\times 3} & {}_{O_{ip}}^{O_{p}}R^{T} \end{array}\right]\left[\begin{array}{c} \begin{array}{c} \begin{array}{c} \begin{array}{c} \Delta x_{i}\\ \Delta y_{i} \end{array}\\ \Delta z_{i}\\ \Delta \alpha_{x_{i}} \end{array}\\ \Delta \alpha_{y_{i}} \end{array}\\ \Delta \alpha_{z_{i}} \end{array}\right]=J_{i}\Delta X_{i}$$

where Δ***X*** is displacement vector at the reference point of the upper platform.

According to the synthesis principle of the space force system, the relationship between the external force vector at the reference point of the upper platform and the reaction force vector on the six flexible series branches is established by the following expression:

(22) $$F=\left[\begin{array}{c} \begin{array}{c} \begin{array}{c} \begin{array}{c} \begin{array}{c} f_{x}\\ f_{y} \end{array}\\ f_{z} \end{array}\\ m_{x} \end{array}\\ m_{y} \end{array}\\ m_{z} \end{array}\right]=\sum_{i=1}^{6}\left(\left[\begin{array}{cc} {}_{O_{ip}}^{O_{p}}R & 0_{3\times 3}\\ S\left(r_{i}\right){}_{O_{ip}}^{O_{p}}R & {}_{O_{ip}}^{O_{p}}R \end{array}\right]\left[\begin{array}{c} \begin{array}{c} \begin{array}{c} \begin{array}{c} \begin{array}{c} f_{xi}\\ f_{yi} \end{array}\\ f_{zi} \end{array}\\ m_{xi} \end{array}\\ m_{yi} \end{array}\\ m_{zi} \end{array}\right]\right)=\sum_{i=1}^{6}J_{F_{i}}F_{i}$$

According to the definition of stiffness matrix of flexible parallel mechanism, there is:

(23) $$F=K\Delta X=\sum_{i=1}^{6}J_{F_{i}}F_{i}=\sum_{i=1}^{6}J_{F_{i}}K_{i}\Delta X_{i}=\sum_{i=1}^{6}J_{F_{i}}K_{i}J_{i}^{-1}\Delta X$$

The stiffness matrix of the upper platform reference point can be obtained by the above equation:

(24) $$K=\sum_{i=1}^{6}J_{F_{i}}K_{i}J_{i}^{-1}$$

The relationship between the end force of each flexible element and the end force of the leg is:

(25) $$F_{ij}=J_{Fij}F_{i}$$

The relationship between the end force of each flexible element and the six-axis external force acting on the sensor’s upper platform is:

(26) $$F_{ij}=J_{Fij}K_{i}J_{i}^{-1}K^{-1}F$$

The relationship between the six forces expressed in the local coordinate system and the six external forces acting on the sensor platform is:

(27) $$F_{ij}^{O_{ip}}=J_{O_{ip}}J_{Fij}K_{i}J_{i}^{-1}K^{-1}F$$

For Equations (21)–(27), there are: *i* = 1,2,…,6; *j* = 1,…,4.

By extracting equations when *j* = 2, it can be obtained:

(28) $$\left[\begin{array}{c} f_{12x}^{O_{1p}}\\ f_{22x}^{O_{2p}}\\ \vdots \\ f_{62x}^{O_{6p}} \end{array}\right]=\left[\begin{array}{c} {\left(J_{O_{1p}}J_{12}K_{1}J_{1}^{-1}K^{-1}\right)}_{1:}\\ {\left(J_{O_{2p}}J_{22}K_{2}J_{2}^{-1}K^{-1}\right)}_{1:}\\ \vdots \\ {\left(J_{O_{6p}}J_{62}K_{6}J_{6}^{-1}K^{-1}\right)}_{1:} \end{array}\right]\left[\begin{array}{c} f_{x}\\ f_{y}\\ f_{z}\\ m_{x}\\ m_{y}\\ m_{z} \end{array}\right]$$

That is *f* = [$G_{F}^{f}]'F$, [$G_{F}^{f}]'$ is the mapping matrix from the external force/toque applied on the measuring platform of the sensor to the force produced on the six elastic legs considering elastic deformation of the flexible joints.

### 3.3. Comparative Analysis of Numerical Simulation and Mathematical Models

**Figure 9:**
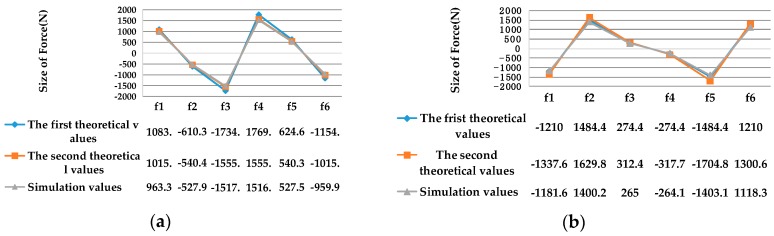

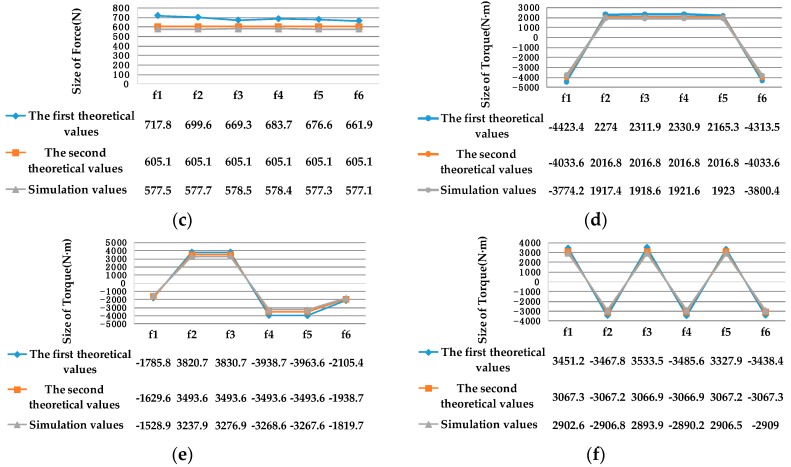
(**a**) Comparison of theoretical values and simulation values loading force along X-axis; (**b**) Comparison of theoretical values and simulation values loading force along Y-axis; (**c**) Comparison of theoretical values and simulation values loading force along Z-axis; (**d**) Comparison of theoretical values and simulation values loading torque along X-axis; (**e**) Comparison of theoretical values and simulation values loading torque along Y-axis; (**f**) Comparison of theoretical values and simulation values loading torque along Z-axis.

In the above two sections, the mathematical models of the two cases are obtained. By using the ANSYS finite element simulation software platform, the external force ***F***_1_ = [5000 0 0 0 0 0 ]^T^, ***F***_2_ = [0 5000 0 0 0 0]^T^ , ***F***_3_= [0 0 5000 0 0 0]^T^ , ***F***_4_ = [0 0 0 5000 0 0]^T^, ***F***_5_ = [0 0 0 0 5000 0]^T^, ***F***_6_ = [0 0 0 0 0 5000]^T^ are respectively loaded in the geometry center of the measuring platform in the three-dimensional model of the sensor, and the size of the force produced on the six elastic legs are obtained by the simulation calculation. The accuracy comparison of the theoretical values and simulation values is shown in Figure 9 and Table 1. In the ideal case, the theoretical value of the mathematical model is recorded as the first theoretical value, and the theoretical value considering elastic deformation of flexible joints is recorded as the second theoretical values.

As shown in Table 1, the measurement errors of the six elastic legs are reduced to 10% after considering the deformation stiffness error of flexure joints, which proves that the mathematical model is effective. 

## 4. Calibration Experiment

### 4.1. Prototype

**Figure 10:**
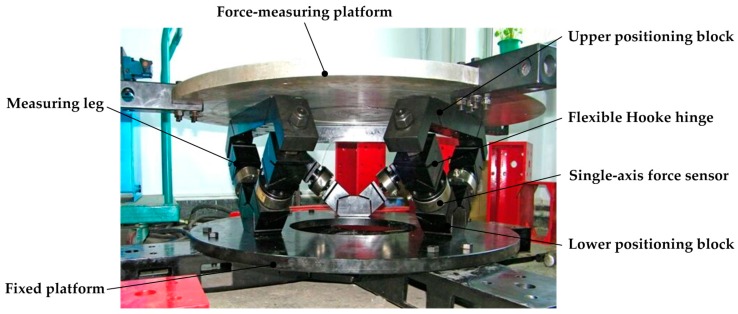
Prototype of the large measurement range six-axis force sensor of 6-UPUR parallel mechanism with flexible joints.

In order to prove the superiority of the proposed sensor structure and prove the correctness of the theoretical analysis, a prototype of the large measurement range six-axis force sensor of 6-UPUR parallel mechanism with flexible joints was manufactured, as shown in Figure 10. Considering the manufacturing process and economic cost, the material property of each component was selected as shown in Table 2. The main structure parameters and the measuring range of the sensor are shown in Table 3 and Table 4. 

### 4.2. Calibration Experiment

**Figure 11:**
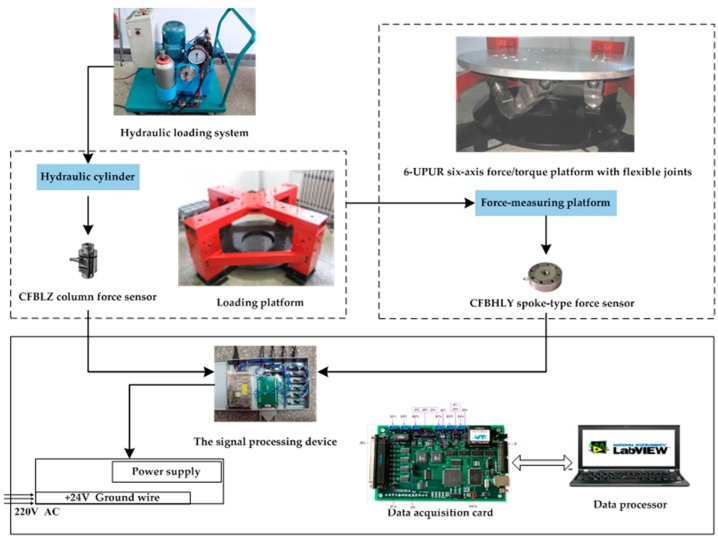
Structure of the calibration experiment system.

**Figure 12:**
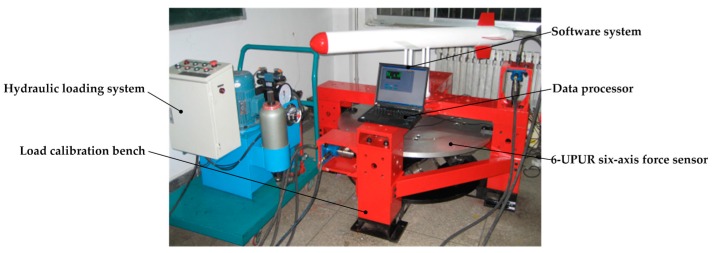
Diagram of the calibration experiment system.

To measure the loaded external force accurately, the sensor should be calibrated by using a special calibration system, which can generate forces and torques in directions of *x, y, z* separately. In this paper the calibration system, which mainly included a hydraulic loading system, calibration platform, parallel 6-UPUR six-axis force sensor with flexible joints, signal processing device, data acquisition device, data processor, calibration software system and so on, as shown in Figure 11 and Figure 12, was designed and manufactured.

Considering that the structure of the six-axis force sensor of 6-UPUR parallel mechanism with flexible joints is very complex and the presence of measuring error can’t be ignored because of the influence of these factors such as the design principles, manufacturing and processing errors, so multiple point loading in the sensor range and the method of least squares linear fit are needed in calibration experiments. Thus, the linear relationship between inputs and outputs of the large measurement range 6-axis force sensor of 6-UPUR parallel mechanism with flexible joints can be calculated.

**Figure 13:**
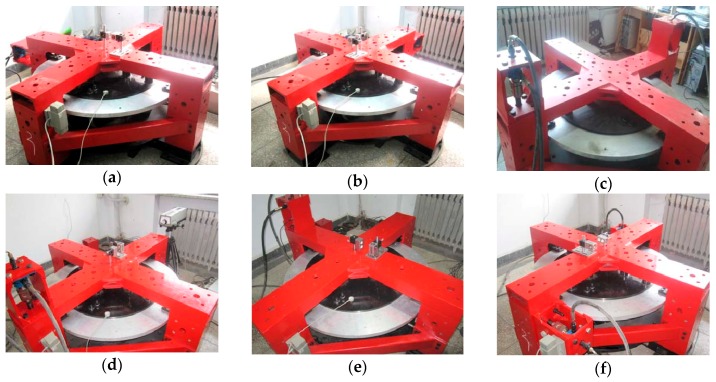
(**a**) Force loading along x-axis; (**b**) force loading along y-axis; (**c**) force loading along z-axis; (**d**) torque loading around x-axis; (**e**) torque loading around y-axis; (**f**) torque loading around z-axis.

Detailed experimental steps are described are as follows:

(1) Each axis force/torque within the sensor range is divided into 10 load points in two positive and negative directions, as shown in Table 5;
(2) Select the force/torque component of the load. The load unit is installed in the corresponding position, and connect the calibration hardware system, as shown in Figure 13;
(3) At each loaded point, conduct loading and unloading experiments in turn, and record the output voltage of each measuring leg corresponding to each load point;
(4) Repeat Steps (2) and (3), conduct loading and unloading experiments in the opposite direction and record the experimental data;
(5) Repeat Steps (2)–(4), the calibration experiments of the six axis force sensor are carried out and all the experimental data are obtained;
(6) Check and process the data. The calibration matrix of the sensor is obtained, and the linearity of the sensor is determined.

### 4.3. Experimental Method

In the analysis of experimental data, the forces that are loaded on the prototype during the experiment can be described as six linearly independent vectors, denoted as:

(29) $$F_{S}=\left[\begin{array}{cccccc} F_{x} & 0 & 0 & 0 & 0 & 0\\ 0 & F_{y} & 0 & 0 & 0 & 0\\ 0 & 0 & F_{z} & 0 & 0 & 0\\ 0 & 0 & 0 & M_{x} & 0 & 0\\ 0 & 0 & 0 & 0 & M_{y} & 0\\ 0 & 0 & 0 & 0 & 0 & M_{z} \end{array}\right]$$

When the external forces are loaded on the measuring platform of the sensor, the six single-axis force sensors will sense the force produced on the measuring legs, and then the change of output voltage of the Wheatstone bridges can be measured out. We can get a set of relationships:

***F**_s_* = ***GV*** (30)

where ***G*** is the calibration matrix between the loaded forces and the output voltages; ***V*** is the output voltage matrix.

Thus the calibration matrix between the loaded forces and the output voltages of the six measuring legs can be calculated:

***G*** = ***F***_s_***V***^−1^ (31)

Besides, the error matrix evaluating the accuracy of the six-axis force sensor is defined as follows:

(32) $$E_{rr}=\frac{F_{S}-F_{6\times 6}}{F_{FS}}$$

where ***F****_FS_* is the full range of the sensor, ***F***_6__×6_ can be calculated based on the mapping matrix between the loaded force and the output voltage by Equation (32); the error matrix ***E****_rr_* is a comprehensive evaluation. The diagonal components of the error matrix separately represent the measurement errors of the six different directions of the loaded external force, and other components represent the interference errors between different directions.

## 5. Experiment Results

### 5.1. Calibration Results and Analysis

Through the calibration system described above, the calibration experiments based on the sensor prototype are carried out, and the data of the six measuring legs are obtained. Taking a complete static calibration experimental data and using the K value method and least square method to substitute the loading forces and the voltages data which are collected from six measuring legs into the corresponding equation, the static calibration matrix ***G****_K_*, ***G***_2*C*_ and the error matrix ***E****_rrK_*, ***E****_rr_*_2*C*_ can be obtained by the analysis and process of the experimental data, therefore, the performance of force-measuring of the sensor is obtained:

(33) $$G_{K}=\left[\begin{array}{cccccc} -0.0694 & -0.2151 & 0.2443 & -0.4298 & -0.5077 & 0.2468\\ -0.5148 & 0.4170 & 0.2045 & -0.2858 & 0.2491 & -0.1535\\ 0.2528 & 0.2198 & 0.2476 & 0.2298 & 0.2288 & 0.2653\\ -0.1336 & 0.1129 & 0.1250 & 0.0064 & -0.0033 & -0.1244\\ -0.0807 & -0.0332 & -0.0452 & 0.1647 & 0.1822 & -0.0460\\ -0.1247 & 0.0999 & -0.0879 & 0.0881 & -0.1108 & 0.0831 \end{array}\right]$$

(34) $$E_{rrK}=\left[\begin{array}{cccccc} 0.0431 & 0.0240 & 0.0249 & 0.0054 & 0.0117 & 0.0093\\ 0.0279 & 0.0264 & 0.0118 & 0.0083 & 0.0082 & 0.0556\\ 0.0607 & 0.0216 & 0.0333 & 0.0141 & 0.0038 & 0.0062\\ 0.0939 & 0.0291 & 0.0270 & 0.0208 & 0.0230 & 0.0135\\ 0.0956 & 0.1340 & 0.0295 & 0.0252 & 0.0163 & 0.0036\\ 0.0519 & 0.0174 & 0.0696 & 0.0216 & 0.0219 & 0.0076 \end{array}\right]$$

From Equation (34), it can be noted that when the K value method is used to decouple, the calibration error of each axis is: ***F****_x_* (4.31%), ***F****_y_* (2.64%), ***F****_z_* (3.33%), ***M****_x_* (2.08%), ***M****_y_* (1.63%), ***M****_z_* (0.76%). The maximum error of the I kind is 4.31%, which appears in ***F****_x_* ; the maximum error of the II kind is 13.40%, which is appears in ***F****_y_*, when loaded in ***M****_y_*. So the calibration error obtained by this method is 13.40%.

(35) $$G_{2C}=\left[\begin{array}{cccccc} -0.0929 & -0.2095 & 0.2377 & -0.4204 & -0.5071 & 0.2437\\ -0.4518 & 0.4031 & 0.1981 & -0.2675 & 0.2288 & -0.1496\\ 0.2546 & 0.2748 & 0.2717 & 0.2628 & 0.2603 & 0.2678\\ -0.1202 & 0.1127 & 0.1251 & 0.0148 & -0.0137 & 0.1157\\ 0.0680 & -0.0508 & -0.0380 & 0.1533 & 0.1703 & -0.0373\\ -0.0877 & 0.0757 & -0.1058 & 0.1067 & -0.1174 & 0.0917 \end{array}\right]$$

(36) $$E_{rr2C}=\left[\begin{array}{cccccc} 0.0122 & 0.0068 & 0.0124 & 0.0026 & 0.0070 & 0.0071\\ 0.0144 & 0.0066 & 0.0111 & 0.0022 & 0.0038 & 0.0061\\ 0.0072 & 0.0062 & 0.0089 & 0.0059 & 0.0028 & 0.0039\\ 0.0169 & 0.0088 & 0.0203 & 0.0039 & 0.0068 & 0.0063\\ 0.0167 & 0.0268 & 0.0074 & 0.0049 & 0.0059 & 0.0038\\ 0.0070 & 0.0061 & 0.0112 & 0.0047 & 0.0020 & 0.0042 \end{array}\right]$$

From Equation (36), it can be noted that when the least squares method is used to decouple, the calibration error of each axis is: ***F****_x_* (1.22%), ***F****_y_* (0.66%), ***F****_z_* (0.89%), ***M****_x_* (0.39%), ***M****_y_* (0.59%), ***M****_z_* (0.42%).The maximum error of the I kind is 1.22%, which appears in ***F****_x_*; the maximum error of the II kind is 2.68%, which appears in ***F****_y_*, when loaded in ***M****_y_*, so the calibration error obtained by this method is 2.68%.

From the results of the calibration experiment, the calibration error comparison of the two kinds of decoupling methods is as shown in Table 6. The results show that the accuracy of least squares method is much better than that of the K value calibration method.

### 5.2. Linearity Analysis

The input signal and the output signal of the sensor are not completely linear, and there is always an error. The ratio of the error to the measurement range is called the linearity of the sensor. When a certain force/torque is loaded to one direction of the sensor, the output voltage of the six legs will change with the change of the loading force/torque. Figure 11 shows the changing curves between the sensor’s measuring force and the standard loading force when the force or torque is loaded in one direction.

**Figure 14:**
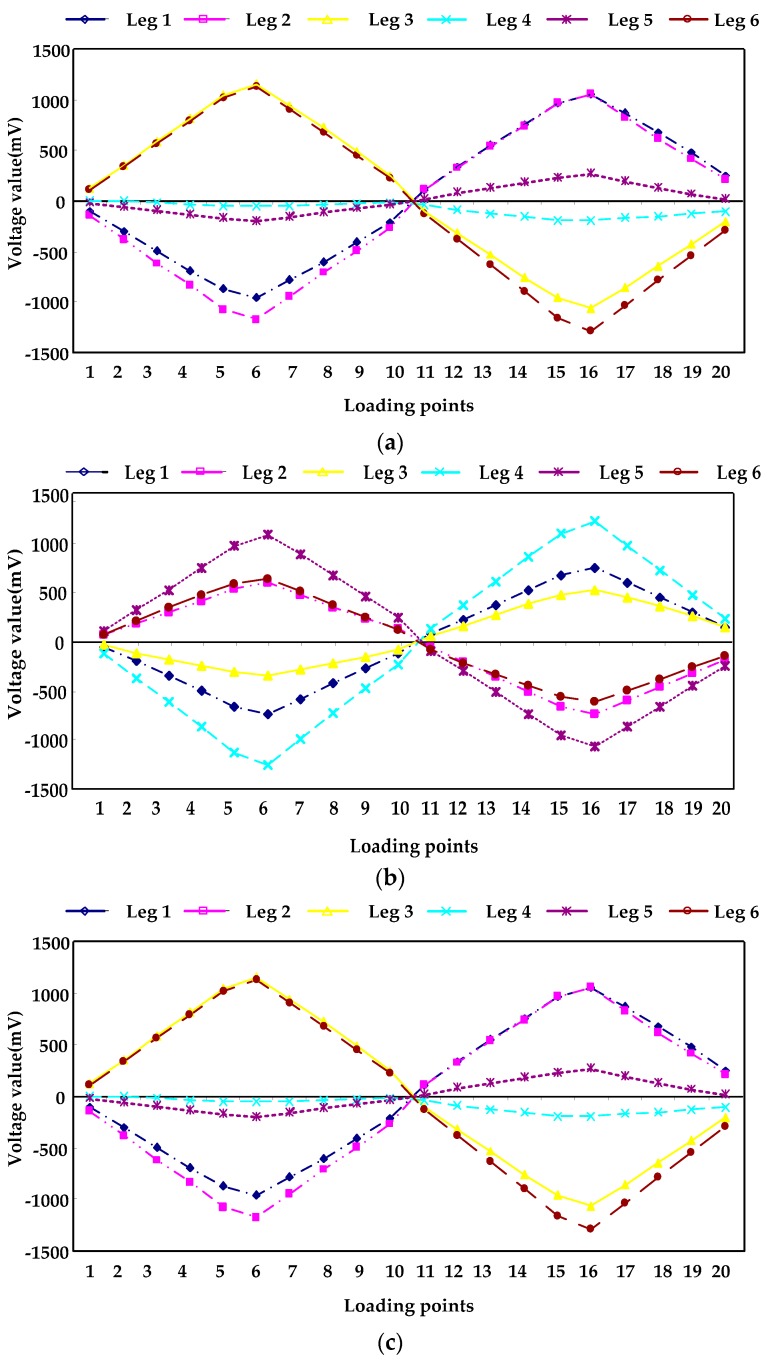
(**a**) Voltage curves of legs loading force along X-axis; (**b**) Voltage curves of legs loading force along Y-axis; (**c**) Voltage curves of legs loading force along Z-axis.

**Figure 15:**
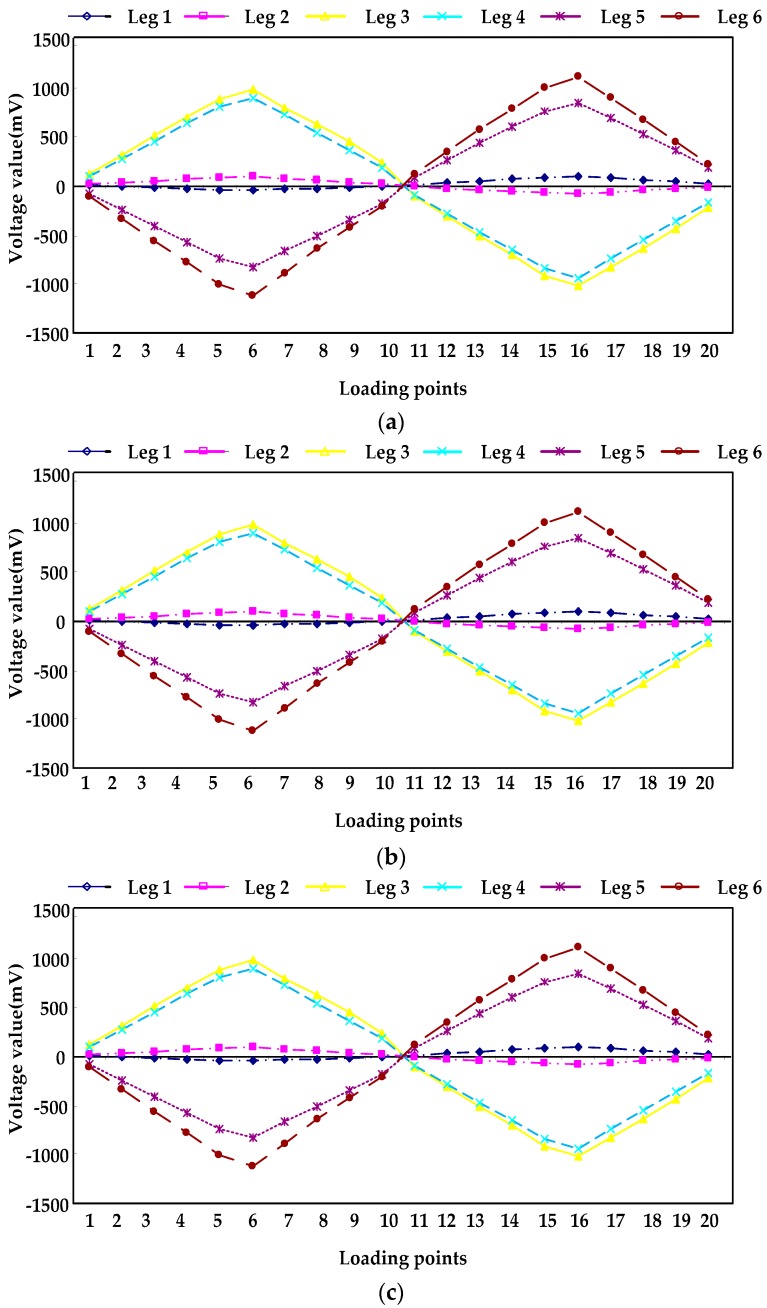
(**a**) Voltage curves of legs loading torque along X-axis; (**b**) Voltage curves of legs loading torque along Y-axis; (**c**) Voltage curves of legs loading torque along Z-axis.

Figure 14 and Figure 15 are the variations of the voltage of each leg with the loading force/torque changing, when the force/torque is loaded in the three directions of X, Y and Z.

Table 7 is the linearity of each leg obtained by least square method. As shown in Table 6, the linearity of each leg of the sensor is less than 1%, which shows that the sensor has a good linearity.

## 6. Conclusions

In this paper, to overcome the influence of the gap and friction of the traditional joints on the parallel six-axis sensors’ precision and stability, we have successfully demonstrated a kind of six-axis force sensor based on 6-UPUR parallel mechanism with flexible joints, which has large measurement range and high accuracy. The force mathematical model of the sensor is established on the screw theory; according to the relations of the stiffness and deformation compatibility condition, the stiffness matrix considering flexibility of each flexible joint is built up; then the complete mathematical model is established. The sensor prototype and the calibration system are manufactured and static calibration experiments were carried out on the sensor. The results show that the measurement error is less than 2.68%, which shows that the sensor has high measuring accuracy and good linearity. The experimental results prove the feasibility of the large measurement range six-axis force sensor of 6-UPUR parallel mechanism with flexible joints. The design and loaded research of the parallel six-axis force sensor has reference significance.

## Figures and Tables

**Table 1:** Error comparison between numerical simulation and mathematical models.

		*F*_1_	*F*_2_	*F*_3_	*F*_4_	*F*_5_	*F*_6_
***F**_x_*	IS^1^(%)	12.5	15.6	14.3	16.7	18.4	20.3
	DS^2^(%)	5.69	2.30	2.46	2.54	2.37	5.47
***F**_y_*	IS(%)	13.2	16.4	17.9	20.3	21.5	16.3
	DS(%)	2.35	5.67	3.42	3.75	5.48	2.27
***F**_z_*	IS(%)	24.3	21.1	15.7	18.2	17.2	14.7
	DS(%)	4.55	4.52	4.39	4.41	4.58	4.62
***M**_x_*	IS(%)	17.2	18.6	20.5	21.3	12.6	13.5
	DS(%)	6.43	4.93	4.87	4.72	4.65	5.78
***M**_y_*	IS(%)	16.8	18.0	16.9	20.5	21.3	15.7
	DS(%)	6.18	7.32	6.20	6.44	6.47	6.14
***M**_z_*	IS(%)	18.9	19.3	22.1	20.6	14.5	18.2
	DS(%)	5.37	5.23	5.64	5.76	5.24	5.16

IS^1^ (%) is the error between the theoretical and the simulation values in the ideal state; DS^2^ (%) is the error between the theoretical and the simulation values in the case of considering the deformation stiffness of flexure joints.

**Table 2:** Material properties of the components.

Components	Materials	Elastic Modulus	Poisson Ratio	Density
Force-measuring platform	Hard aluminum alloy	70 Gpa	0.30	2700 kg/m^3^
Flexible joints	40CrNiMoA	206 Gpa	0.30	7830 kg/m^3^
Fixed platform	Q235	210 Gpa	0.25	7850 kg/m^3^

**Table 3:** Structure parameters of six-axis force sensor.

A (°)	B (°)	C (°)	R (mm)	R (mm)	*l*_1_ (mm)	*l*_2_ (mm)
65.6	40.0	40.0	466.7	300.0	300.0	163.6

**Table 4:** Measuring range of the six-axis force sensor.

*F_x_* (N)	*F_y_* (N)	*F_z_* (N)	*M_x_* (N·m)	*M_y_* (N·m)	*M_z_* (N·m)	Overload Capacity
±10,000	±10,000	±10,000	±5000	±5000	±5000	120%

**Table 5:** Loading points of calibration force/torque.

Loading Points		1	2	3	4	5	6	7	8	9	10
**Force (N)**	**Positive**	1000	2000	3000	4000	5000	6000	7000	8000	9000	10,000
	**Negative**	10,000	9000	8000	7000	6000	5000	4000	3000	2000	1000
**Torque (N·m)**	**Positive**	500	1000	1500	2000	2500	3000	3500	4000	4500	5000
	**Negative**	5000	4500	4000	3500	3000	2500	2000	1500	1000	500

**Table 6:** The calibration error comparison of the two kinds of decoupling methods.

Decoupling Method	Type I Error (%)							Type II Error (%)
	*F_x_*	*F_y_*	*F_z_*	*M_x_*	*M_y_*	*M_z_*	Maximum Value	Maximum Value
K value method	4.31	2.64	3.33	2.08	1.63	0.76	4.31	13.40
Least squares method	1.22	0.66	0.89	0.39	0.59	0.42	1.22	2.68

**Table 7:** Linearity of six-axis force sensor.

Items	Linearity (%)					
	Leg 1	Leg 2	Leg 3	Leg 4	Leg 5	Leg 6
***F**_x_*	0.22	0.18	0.23	0.24	0.08	0.15
***F**_y_*	0.20	0.14	0.29	0.22	0.13	0.21
***F**_z_*	0.14	0.18	0.71	0.64	0.57	0.97
***M**_x_*	0.21	0.19	0.82	0.32	0.39	0.92
***M**_y_*	1.00	0.75	0.51	0.24	0.4	0.4
***M**_z_*	0.23	0.39	0.25	0.15	0.15	0.39

## Abbreviations

6-UPUR	A kind of mechanism configuration. U-universal pair, P-prismatic pair, R-revolute pair.
LVDT	linear variable differential transformer.
